# Neurocognitive mechanisms of statistical-sequential learning: what do event-related potentials tell us?

**DOI:** 10.3389/fnhum.2014.00437

**Published:** 2014-06-18

**Authors:** Jerome Daltrozzo, Christopher M. Conway

**Affiliations:** Department of Psychology, Georgia State UniversityAtlanta, GA, USA

**Keywords:** sequential learning, statistical learning, implicit learning, procedural learning, artificial grammar, ERP, P300, P600

## Abstract

Statistical-sequential learning (SL) is the ability to process patterns of environmental stimuli, such as spoken language, music, or one’s motor actions, that unfold in time. The underlying neurocognitive mechanisms of SL and the associated cognitive representations are still not well understood as reflected by the heterogeneity of the reviewed cognitive models. The purpose of this review is: (1) to provide a general overview of the primary models and theories of SL, (2) to describe the empirical research – with a focus on the event-related potential (ERP) literature – in support of these models while also highlighting the current limitations of this research, and (3) to present a set of new lines of ERP research to overcome these limitations. The review is articulated around three descriptive dimensions in relation to SL: the level of abstractness of the representations learned through SL, the effect of the level of attention and consciousness on SL, and the developmental trajectory of SL across the life-span. We conclude with a new tentative model that takes into account these three dimensions and also point to several promising new lines of SL research.

## INTRODUCTION

From an ecological point of view, learning about temporal patterns in our environment, and using this information to make predictions about upcoming events and actions, is arguably of primary importance to humans and other higher-order organisms (Lashley, 1951; Conway et al., 2010; Goldstein et al., 2010). In the past 15 years, an increasingly established body of research has demonstrated that humans have a remarkable ability to learn statistical patterns – i.e., commonalities and underlying regularities – from among a set of stimuli, a phenomenon now reffered to simply as “statistical learning” (Saffran et al., 1996, 1997). A related phenomenon, known as “implicit learning,” likewise reveals people’s ability to learn predictive patterns without conscious intent or awareness (Cleeremans and McClelland, 1991; Berry and Dienes, 1993). Both statistical learning and implicit learning have been observed with many different types of input materials in sensory (e.g., music, speech, and visual patterns) and motor domains. In fact, due to the apparent commonalities between statistical learning and implicit learning, there is growing consensus that these two phenomena may actually tap into the same process (Perruchet and Pacton, 2006).

In the current review, we focus in particular on the learning of temporal or sequential patterns of stimuli and therefore use the term “statistical-sequential learning” or simply “sequential learning” (SL) for short. Because it is still an open question as to whether these learning abilities are also governed at least in part by explicit processes (e.g., Baddeley and Wilson, 1994; Cleeremans, 2006; Haider and Frensch, 2009; Jamieson and Mewhort, 2009; Dale et al., 2012), we avoid the use of the term “implicit” (and in subsequent sections we directly address the different contributions of implicit and explicit processes). Under this definition, SL is the ability to learn underlying structured patterns that exist among a set of non-random, sequentially presented stimuli (Conway and Christiansen, 2001; Conway, 2012). Yet another term recently used that also captures this crucial aspect of statistical-sequential learning is “structured sequence processing” (Uddén and Bahlmann, 2012).

To date, the underlying cognitive and neural mechanisms of SL and the associated cognitive representations are still not well understood. SL has been explored though a combination of cognitive modeling and empirical studies using behavioral and neurophysiological measurements. The current outcome of these heterogeneous approaches is that the proposed theories of SL still need to be confirmed by empirical evidence. The purpose of this review is to provide an initial assessment of the current theories of SL and to identify the areas of empirical research that need further development. Due to the extensive behavioral and neural SL literature, the scope of this review will focus on the exploration of SL with a specific neural approach, the event-related potential (ERP) technique (for other neuroimaging techniques, see for example Seger et al., 2000; Bischoff-Grethe et al., 2001; Huettel et al., 2002; Skosnik et al., 2002; Lieberman et al., 2004; Petersson et al., 2004; Thomas et al., 2004; Forkstam et al., 2006; Turk-Browne et al., 2009; Uddén and Bahlmann, 2012).

Since SL has been observed in multiple modalities and domains, we draw upon a wide range of empirical studies, reviewing for instance studies on motor learning, visual-motor learning, visual-perceptual learning, auditory learning of different types of stimuli, language learning, and social learning. Recognizing the differences across these studies when relevant, we also focus on the commonalities among them in order to bring to light what we believe is the cognitive process at the core of all of them.

We first summarize the primary theoretical views of SL. We then review the main approaches used by ERP research to study SL. This will point to a discrepancy between the theoretical and the empirical approaches, highlighting a series of fundamental unanswered questions. Finally, we provide suggestions for moving forward to address the most challenging aspects of SL research and provide a tentative new model of SL that incorporates much of the existing empirical and theoretical advances.

## MODELS AND THEORIES OF SEQUENTIAL LEARNING

Three primary questions about the nature of SL have intrigued researchers over the decades, organized around a limited set of non-orthogonal (i.e., partly overlapping) dimensions: (1) the extent to which SL encodes and manipulates concrete versus abstract representations, (2) whether SL depends on the level of conscious awareness or attention, and (3) how SL changes across the life-span. We consider each of these issues in turn.

### CONCRETE VERSUS ABSTRACT REPRESENTATIONS

Sequential learning could in principal encode either: (1) concrete features of the sequence, such as the frequencies of individual items (or exemplars) of the sequence, (2) or abstract features, e.g., abstract rule(s) that organize the to-be-learned sequence (Franco and Destrebecqz, 2012). This section refers primarily to the types of representations that are manipulated by the SL mechanism(s).

Reber (1967) – using an artificial grammar paradigm – was the first to propose that SL is the result of the implicit learning of abstract rules. This proposal was later endorsed by several others (e.g., McAndrews and Moscovitch, 1985; Mathews et al., 1989; Dienes et al., 1991; Knowlton et al., 1992; Knowlton and Squire, 1993, 1994, 1996; Manza and Reber, 1997; Marcus et al., 1999; Rossnagel, 2001; Kuhn and Dienes, 2005). The idea that the cognitive system was able to unconsciously process abstract information, the so-called “smart unconscious” hypothesis (Cleeremans et al., 1998), was for many researchers somewhat provocative and was challenged by connectionist computational modeling (Christiansen et al., 1998). Connectionist models showed that rather than the learning of abstract rules, several results of the SL literature could be successfully modeled using only concrete feature processing, such as the processing of chunks or transitional probabilities (Perruchet and Pacton, 2006).

Perhaps the best-known empirical demonstration of SL comes from Saffran et al. (1997), who used a word segmentation task in which a continuous sequence of syllables was presented (e.g., “bupadapatubitutibu”). The syllable sequence covertly consisted of artificial “words” (e.g., “bupada” and “patubi”) spliced together. Participants demonstrated above-chance performance in a subsequent recognition test, discriminating words from non-word syllable groupings. Saffran et al. (1997) proposed that such performance was achieved by exploiting the statistical regularities present in the sequence of syllables, such as transitional probabilities between successive syllables (e.g., the probability that a given syllable A is immediately followed by another given syllable B) that are higher within words than between words. These statistical regularities are one type of concrete feature that could be learned in a sequence.

**FIGURE 1:**
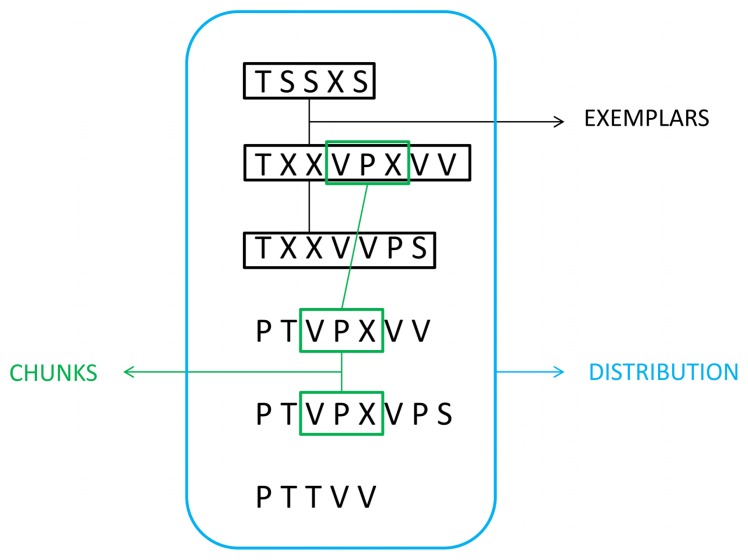
**Three types of concrete feature representations involved in encoding a sequence of letter strings generated from an artificial grammar (see “Artificial Grammar and Natural Language Paradigms” section): fragment-based or chunk information, exemplars, and distributional information (modified with permission from Cleeremans et al., 1998)**.

The acquisition of these concrete features is often referred to as “surface learning” or “fragmentary learning” (Perruchet and Pacteau, 1990; Servan-Schreiber and Anderson, 1990; Perruchet and Amorim, 1992; Meulemans and Van der Linden, 1997). Surface learning may be based on the encoding of item frequencies and item variability across the sequence (Maye et al., 2002; Perruchet et al., 2004; Clayards et al., 2008). Cleeremans et al. (1998) reviewed at least three types of concrete features that once learned could account for many results of the SL literature: fragment-based or chunk information, exemplars, and distributional information (**Figure 1**). In the same vein, several models have been proposed to account for surface learning based on the to-be-learned type of concrete information. Some models focused on conditional statistics between items of the sequence (Thiessen and Pavlik, 2013) and others on the use of temporal contingencies (Montague and Sejnowski, 1994) that may covary in a cause-effect relationship with the physical world (Gopnik et al., 2004).

These concrete feature-based models are computational and have been criticized as such. For instance, the simple-recurrent-network model (Elman, 1990; Cleeremans and McClelland, 1991), has been argued to suffer major weaknesses (McCloskey and Cohen, 1989; Goldstein et al., 2010) with (1) long range dependencies, as in “embedded sequences” (e.g., Uddén and Bahlmann, 2012); (2) sequences made of large sets of rules and items of the scale found in natural language, notably because they are designed to consider the entire corpus of input simultaneously, rather than in the proper temporal order (Goldstein et al., 2010); and (3) multimodal data (Goldstein et al., 2010).

Related to the issue of abstractness, SL could result in modality-specific (more concrete) or amodal (more abstract) representations. For Reber, SL was a mainly amodal process (Reber, 1989); however, some research has suggested that both domain-general (Clegg et al., 1998; Kirkham et al., 2002; Bapi et al., 2005) and modality-specific SL might coexist (Keele et al., 2003; Conway and Christiansen, 2005; Conway and Pisoni, 2008; Turk-Browne et al., 2009; Shafto et al., 2012). For example, Keele et al. (2003) proposed two independent SL systems based on the available behavioral and neuroimaging findings at the time. One system integrates all sequential information regardless of the input modality (presumably relying on more “abstract” representations that are not tied to a particular input modality), while a second system captures only the patterns of a sequence within a single modality (more reliant on “concrete” or modality-specific representations), without suffering interference from intervening sequential information from other modalities. Keele et al.’s (2003) two-system model of SL is therefore consistent with the notion that SL might encode both concrete (stimulus-specific) and more abstract (domain-general) patterns.

Some models of SL in fact explicitly incorporate a multilayer structure. Clegg et al. (1998) suggested three levels of processing: (1) an abstract level storing higher-level goals that are neither stimulus- nor response-related; (2) an intermediate level encoding the type of action required (independently of the effector) or the stimulus specificity (independently of its exact identity); and (3) a low level acquiring highly specific information related to the exact stimulus and the associated final motor execution. Possibly a parallel could be drawn between the representations processed by these three layers and the concrete-abstract continuum. Multilayer models like Clegg et al. (1998) have the advantage of providing an account of both concrete feature learning and more abstract situations, such as the “transfer of learning” paradigm, which indicates that the representation of a sequence may not be tied to a particular effector or stimulus domain (Clegg et al., 1998).

Related to the issue of modality-specificty, it should be noted that the more concrete-based aspects of SL appear to show similarities to perceptual learning (PL), which allows for the development of spatio-temporal representations of the environment through learning along various levels of cortical processing (Sagi and Tanne, 1994; Skrandies and Fahle, 1994; Goldstone, 1998; Conway et al., 2007). Interestingly, PL and perceptual-based SL seem to activate similar neural networks (Turk-Browne et al., 2009). Like SL, PL can occur with rather short exposure to patterns, can have long lasting effects, and can occur without attention to or awareness of the patterns; however, PL can also be modulated by levels of attention and awareness (Goldstone, 1998; Alain et al., 2007; Sasaki et al., 2010; Lu et al., 2011; Aberg and Herzog, 2012; Byers and Serences, 2012; Kumano and Uka, 2013). Furthermore, PL is, like SL, often described as being at the root of language learning, particularly for the development of phonological and lexical representations (Goldstone, 1998; Cutler, 2008; Samuel and Kraljic, 2009; Werker, 2012) and is also proposed as a process required for motor preparation and execution (Hommel et al., 2001). According to a standard definition of SL – the ability to learn patterns of stimuli unfolding in time – SL can be seen as the “temporal” subcategory of a the more general “spatio-temporal” PL, in which items frequently co-occurring in time (but not spatially) can form new perceptual “units” (Goldstone, 1998). If SL is viewed from this perspective, the development of concrete representations during SL could be explained in terms of properties of PL. Indeed, (temporal) statistical contingencies between items/percepts (e.g., transitional probabilities or perceptual units of co-occuring informations such as chunks, Czerwinski et al., 1992; Seriès and Seitz, 2013) could be captured and stored in cortical spatio-temporal representations.

One final way that, together with abstractness and modality, SL representations might be differentiated is by the types of input structures (Conway and Christiansen, 2001; Conway, 2012). Three types have been proposed: fixed patterns (i.e., invariant or repeating sequences); statistical patterns (sequences containing statistical regularities or distributional information across exemplars); and hierarchical patterns (i.e., embedded sequences with non-adjacent or self-recursive structures). Different neurocognitive mechanisms may be used in the service of each type of input structure (Bahlmann et al., 2006; Uddén and Bahlmann, 2012). These three types of input structures appear related to the concrete-abstract continuum: learning an invariant fixed pattern or statistical regularity is likely represented in a concrete fashion, whereas learning a self-recursive structure is likely represented more abstractly, allowing for generalization of the recursive rule to new exemplars.

It appears likely then that SL involves multiple processes, some that could be characterized as being more domain-general and that manipulate rather abstract representations, and others that are more input-specific and that encode more concrete features. This perspective is similar to the “more-than-one-mechanism” (MOM) hypothesis of language acquisition, stating that language is acquired via the manipulation of both rule-based and statistical representations (Endress and Bonatti, 2007). Several recent models of SL now combine feature-based learning with more abstract forms of rule-learning mechanisms. For instance, Pierrehumbert (2003) provided a model of how abstract rules could be extracted from a speech signal through the interaction between different high and low-level cognitive systems, including bottom-up processing of low-level acoustic and articulatory features. In this model, a phonological system would refine internal categorizations in its different levels through: (1) internal feedback mechanisms from higher level internal systems to lower levels internal systems, and (2) external feedback due to the interaction with the speech community.

The issue of the abstractness of the representations manipulated during SL is complex. Perhaps the most promising accounts of SL involve the processing of both concrete and abstract information (e.g., Clegg et al., 1998; Keele et al., 2003; Pierrehumbert, 2003). The exact interplay among these postulated processes remains unknown and opened to multiple model implementations. In a rather simple model, the two hypothetical mechanisms would work in parallel. One would encode and store modality-specific concrete features in a given format and another mechanism would encode and store domain-general abstract information in another format. A second and perhaps more neurally plausible possibility is a cascading account, whereby the two mechanisms interact in a hierarchical manner, with concrete information being first encoded in a modality-specific format, followed, upon further processing or exposure to the input, by the development and encoding of more abstract and domain-general representations. Accordingly SL across input modalities (e.g., learning that a particular tone predicts a visual stimulus) would present a greater processing challenge than SL within an input modality (e.g., learning that a particular visual stimulus predicts another visual stimulus). This is, in fact, what recent findings appear to indicate (Walk and Conway, 2011).

### IMPLICIT AND EXPLICIT MECHANISMS

In addition to dissociating the mechanisms of SL by the level of abstractness of the learned features, the level of attention (and consciousness) has also been recognized as a critical dimension of SL. The SL literature often refers to this issue in terms of “implicit” and “explicit” processing. Traditionally, SL is generally thought to involve the activation of incidental/implicit, automatic, and even unconscious processes (e.g., Saffran et al., 1996, 1997; Fiser and Aslin, 2001, 2002; Shanks and Perruchet, 2002; Turk-Browne et al., 2005; Shanks et al., 2006; Hannula and Ranganath, 2009; Rosenthal et al., 2010). Several empirical strands of research on SL have suggested that the level of awareness is irrelevant to SL performance (Curran and Keele, 1993; Goschke, 1998; Song et al., 2007). Clegg et al. (1998) not only acknowledge this implicit component of SL but go further by suggesting that SL does not manipulate explicit knowledge representations. Rather, they suggest that explicit knowledge emerges through the interaction of SL with other cognitive systems that can access and modify explicit memories (Clegg et al., 1998).

Alternatively, other theories have argued for a more direct role of explicit processing in SL. For instance, Cleeremans (2006) suggested that a representation obtained from exposure to a sequence may become explicit when the strength of activation of this representation reaches a critical level. Similarly, explicit knowledge may emerge as the result of a search process that is triggered by unexpected events occuring during task processing and requiring an explanation (the unexpected-event hypothesis; Haider and Frensch, 2009). Some authors go even further by drawing a link between “general” consciousness/awareness (i.e., not only of sequence representations) and SL. Dale et al. (2012) proposed that predictive mechanisms such as those that are thought to account for SL may be at the root of the formation of conscious percepts or awareness (Morsella, 2005).

Between these two extreme views there exist proposals that acknowledge the development of both conscious and unconscious representations resulting from SL as well as the contribution of explicit and implicit mechanisms to SL. For instance, Baddeley and Wilson (1994), who analyzed the effect of explicit versus implicit learning in amnesic patients, suggested that implicit learning is strongly dependent on the efficiency of explicit learning, as the later would monitor errors while the former would be heavily impaired by errors during learning. Jamieson and Mewhort (2009) reached a similar conclusion. In their model, they suggested that even though SL can occur without the participant’s explicit knowledge of an underlying rule, SL would nevertheless require memory retrieval of association traces between the current stimulus, the response associated with it, and the context provided by the immediately preceding response. Importantly, they underline that this account of SL does not require implicit learning but instead memory retrieval, that may or may not be fully conscious.

Clearly, there is far from a consensus on the question of whether SL is subserved by implicit or explicit mechanisms, or a combination of both. Nevertheless, perhaps the most influential view to date is that both types of mechanisms contribute to SL (e.g., Curran and Keele, 1993). Importantly, this view finds support from neuroimaging data. Physically distinct brain networks, including dorsolateral prefrontal, medial frontal, and more dorsal posterior regions, appear to be activated when subjects become consciously aware of a sequence. These networks are not activated when subjects are unaware of the sequence rules (Grafton et al., 1995). Such results would be consistent with explicit knowledge leading to the use of working memory to process conscious representations of the sequence (Smith and Jonides, 1995), while areas commonly associated with motor control and/or perceptual processing, including motor cortex, primary sensory areas, and subcortical structures in the basal ganglia, would be activated under conditions of implicit learning (for a more complete discussion see Curran, 1998).

From a methodological point of view, one way to explore the extent of explicit and implicit learning in SL paradigms is to use rapid serial visual presentations (RSVP). Kim et al. (2009), for instance, used such a design together with a matching questionnaire to assess explicit learning and concluded that SL was performed though implicit mechanisms. But several critiques can be raised on the ability to assess purely explicit learning through questionnaire assessments. Thus, novel methods have been developed to better dissociate implicit from explicit learning, such as comparisons between direct and indirect tasks or the process-dissociation procedure (Jacoby, 1991). In direct tasks, such as questionnaire assessments or recognition judgments, subjects are explicitly instructed to respond based on their conscious knowledge. In indirect tasks, performance is measured in a manner that does not require conscious choice by the participants. If participants show greater SL as measured by an indirect task compared to a direct task, it is likely that SL occurred without accompanying conscious awareness (Cleeremans et al., 1998). Taking this logic one step further, Jacoby (1991) proposed the process-dissociation procedure as a method for dissociating implicit from explicit learning. This procedure allows one to separate memories acquired intentionally (i.e., consciously) from memories acquired automatically. Franco et al. (2011) applied this method to explore the cognitive mechanism(s) of SL. They found that statistical information acquired through two SL paradigms containing two different artificial grammars of syllables where only transition probabilities differed, can be consciously manipulated to differentiate these artificial languages. That is, the transitional probabilities became to some extent available to consciousness.

Even though these new methods have improved our ability to assess the contribution of the level of consciousness to SL mechanisms, the issue is far from settled. Some researchers still believe that the assessment of consciousness needs further improvements (Dale et al., 2012). Importantly, the debate about the interaction between consciousness and SL performance essentially distinguishes between two aspects of consciousness: the consciousness of the acquired knowledge (e.g., transitional probabilities) resulting from SL (see for instance Franco et al., 2011) and the level of consciousness available or required during the SL process itself, that is, whether learning was intentional or incidental. One recent empirical study incorporated this distinction by using a dual-task paradigm that induced a cognitive load either during an (incidental) encoding phase or during an (explicit) test phase, or both (Hendricks et al., 2013). Interestingly, the results demonstrated differential effects of the dual-task manipulation, impairing performance only during the explicit test phase, that is, during the manipulation of explicit knowledge, but not during the encoding phase. Furthermore, in a transfer condition in which the elements of each sequence were mapped onto a new subset of items, the dual-task condition eliminated SL regardless of whether it occurred during the encoding phase or during the test phase. This finding suggests that SL is largely an implicit process; however, the expression of previously learned knowledge gained through SL during an explicit test as well as the learning of abstract rules appears to require conscious awareness (Hendricks et al., 2013).

In summary, the literature remains highly heterogeneous in terms of the impact of the level of consciousness on SL performance. However, perhaps the most conservative view, similar to that discussed earlier, is that SL might not be governed by a single cognitive mechanism and might not store representations in a single – e.g., unconscious – format. Instead, SL is likely subserved by at least two mechanisms, one that is rather independent of the level of consciousness/attention and results in unconscious representations and one that depends more on attentional resources and leads to more conscious representations. We will see that ERPs can be helpful in testing this assumption.

### DEVELOPMENTAL CONSIDERATIONS

Whether described in terms of the abstractness of the representations or on the consciousness/attentional dimension, SL can hardly be fully investigated without taking into account its developmental trajectory. Although most SL experiments have been performed with young adults, several studies have focused on SL in children (Saffran et al., 1997; Meulemans et al., 1998; Thomas and Nelson, 2001; Vicari et al., 2003; Thomas et al., 2004; Arciuli and Simpson, 2011; Arciuli and von Koss Torkildsen, 2012) and infants (Haith et al., 1988; Haith and McCarty, 1990; Saffran et al., 1996, 2001; Smith et al., 1997; Aslin et al., 1998; Clohessy et al., 2001; Fiser and Aslin, 2002; Shafto et al., 2012). There are also a handful of studies investigating SL in the elderly population (Prull et al., 2000; Dennis et al., 2003; Howard et al., 2004; Aizenstein et al., 2005; Humes and Floyd, 2005; Shea et al., 2006).

Despite the growing body of research that focuses on SL across the life-span, the developmental progression of SL is still largely unknown. The early literature on implicit learning assumed that this cognitive ability was rather independent of age (Reber, 1993), while explicit learning would improve with aging (Schneider and Pressley, 1997; Parkin and Streete, 1988). Later on, this claim of developmental invariance was contradicted in several instances (Mecklenbräuker et al., 2003; Thomas et al., 2004; Barry, 2007; McNealy et al., 2010). In most cases where developmental differences in implicit learning have been found, young adults out-performed children. However, it appears that in at least some instances, the SL mechanisms of juvenile organisms may be more efficient than those of older ones (McNealy et al., 2010; Johnson and Wilbrecht, 2011); in natural language, this is evidenced by the difficulty with which adults acquire a second language (Gordon, 2000) compared to infants who can display efficient bilingual learning skills (Werker, 2012). Some proposals take the somewhat paradoxical stance that cognitive limitations may confer a computational advantage for learning, which may provide an alternative explanation for the presence of sensitive periods in language development (Newport, 1990; Elman, 1993; Conway et al., 2003). Additional research is needed to explore these ideas further.

In terms of how SL abilities develop later in life, the literature from the elderly population points either to no change in old age in the case of deterministic sequences (Howard and Howard, 1989, 1992; Frensch and Miner, 1994; Cherry and Stadler, 1995; Salthouse et al., 1999) but age-related deficits when sequences are probabilistic or have rather complex structures such as long range dependencies (Curran, 1997; Howard and Howard, 1997; Feeney et al., 2002; Howard et al., 2004). According to “the frontal lobe hypothesis of cognitive aging” (Hess, 2005), this deficit could stem from atypical activation of the dorsolateral prefrontal system, resulting in failures to properly represent and maintain context information (Braver et al., 2001), which in turn might be due to reduced working memory performance.

The model of Pierrehumbert (2003) takes clearly into account the developmental aspect. The author proposes that bottom-up mechanisms, including SL mechanisms – that encode concrete features of sequences – would be the main component of speech processing strategy in infants. Later on, with increased exposure to linguistic materials, this strategy would allow the development of categorizations at higher levels of the phonetic system, which in turn, would trigger top-down feedback mechanisms. Consistent with this model, children show evidence of categorization of the speech stream rather early, by age three (Nittrouer, 1996) and Hazan and Barrett (2000) showed that categorization of consonants in minimal pairs such as boat/goat continues to develop between 6 and 12 years. At age 12, such categorizations have still not reached young adult levels. According to Pierrehumbert, these later developments would result from top-down feedback mechanisms within the phonological system requiring a long process of elaboration and refinement. These top-down mechanisms would explain how initial preconscious levels of representation are progressively refined from childhood to adulthood. Such top-down accounts of SL mechanisms imply that low-level mechanisms of SL do not provide a full picture of the SL in adults and require one to take into account interactions between a more “basic” SL mechanism and information received from higher-level systems of the phonological system. Along this line, one may hypothesize the existence of two types of SL mechanisms: a “basic” and an “expert” mechanism. Infants would benefit almost exclusively from the former, while children, adolescents, and young adults would benefit from the latter becoming increasingly developed as age increases into young adulthood. In older adults, however, the “expert” mechanism, presumably drawing upon working memory resources, might show signs of deficiency.

Thus, similarly to the dissociation of mechanisms of SL into explicit and implicit components, and into mechanisms encoding concrete and abstract representations, the Pierrehumbert (2003) developmental account of SL incorporates two systems that develop differentially. Such a multiple mechanism view of SL is consistent with Gervain and Mehler’s (2010) suggestion that a combination of language-specific, perceptual, and statistical learning mechanisms are all necessary for learning language (Gervain and Mehler, 2010). In their ACCESS model, these elements are combined together with social cues to explain language acquisition performance across the early life-span. Some learning mechanisms would work only on short time-scales while others would require the link of information at longer time-scales (Goldstein et al., 2010). Over short time-scales, infants would use surface structure such as transitional probabilities to extract co-located sequences of phonemes from a continuous input (Saffran et al., 1996; Pelucchi et al., 2009). Over longer time-scales, infants may benefit from social cues, such as parents’ use of common grammatical constructions and incorporate them in their own speech (Cameron-Faulkner et al., 2003). Importantly, such developmental models (i.e., involving multiple mechanisms) have received support from neuroimaging data. For instance, Thomas et al. (2004) provided evidence of a maturation of two distinct mechanisms of SL between childhood and adulthood: a process acting on unconscious representations and another that manipulates explicit knowledge.

**FIGURE 2:**
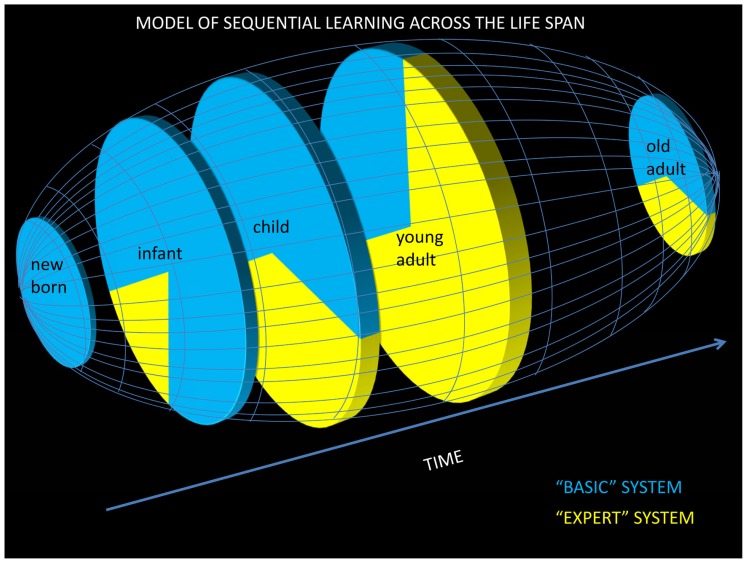
**Model of SL across the life span.** We propose that SL is governed by two systems: a “basic” and an “expert” system. The “basic” system incorporates modality-specific predictive mechanisms that are mostly automatic and implicit and that capture concrete structures of sequences such as chunks and transition probabilities through a bottom-up process. The basic system, which is possibly a sub-system (in the temporal domain) of the (spatio-temporal) PL system, can be modeled by simple recurrent networks. The “basic” system is already available very early in life, allowing for the development of explicit long-term associative memories that become available to the expert SL system. The “expert” system, which relies on top-down explicit multimodal and retrospective mechanisms, depends on the level of intention (to learn) and attention (including selective attention through social cues). The “expert” system, which captures more abstract patterns, increasingly develops from childhood into adulthood and then declines in old age because of impaired working and sensory memories. Blue represents the proportion of SL governed by the basic system and yellow represents the proportion of SL governed by the expert system. Clearly, this model is tentative and highly speculative. In particular, the exact degree of contribution of the basic and expert systems at different ages of life remain currently unknown.

In summary, SL may consist of at least two different systems. The first relies upon bottom-up implicit/perceptual mechanisms that result in unconscious representations, develop early in life, and are likely to exploit surface structure of input and hence can explain some of the impressive language-related abilities present in infants and children. The second system develops later in life, consisting of expert SL mechanisms that rely more on top-down information, are more dependent on the level of attention, and result in explicit knowledge of abstract rules that further improves language processing abilities (but see Marcus et al., 1999, suggesting that abstract information may already be processed by 7-months old as well). Thus, rather than a simple explanation of how a single SL ability progresses over time, it may be necessary to consider at least two different sub-systems and associated mechanisms to draw a complete picture of the developmental trajectory of SL. Understanding how each of these processes develops and interacts dynamically across the life-span remains a formidable research challenge. Based on the preceeding discussion, we propose an initial and albeit simplified model showing the developmental progression of these two SL systems (**Figure 2**). In order to provide extra empirical validation of this model, we now turn to how ERPs have contributed to a better understanding of the mechanisms of SL.

## EXPLORING SEQUENTIAL LEARNING WITH EVENT-RELATED POTENTIALS

**FIGURE 3:**
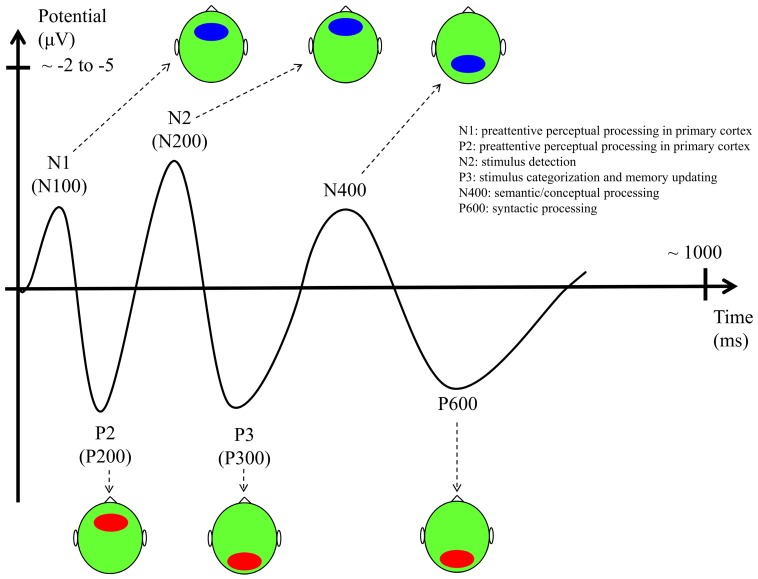
**Main ERP components with their functional interpretation, latencies, and scalp topography (ellipses indicate the scalp location where the component has the largest amplitude – red: positive potential, blue: negative potential; vertical axis unit: scalp potential in microvolts with negativity upward; horizontal axis unit: time from the stimulus onset in milliseconds)**.

We will first summarize the main ERP paradigms that have been used to date in SL research (the main ERP components are described in **Figure 3**). We will then focus on how ERPs have been used to explore the three above-mentioned dimensions of SL mechanisms: the abstractness of the manipulated representation, the level of attention/consciousness of the mechanisms and the level of consciousness of the representations, and the development of SL across the life-span. After considering these three dimensions of SL, we then consider new avenues of research and then conclude with a re-evaluation of the two-system model of SL described in **Figure 2**.

### MAIN ERP PARADIGMS OF SL RESEARCH

#### Oddball and *SRT* paradigms

**FIGURE 4:**
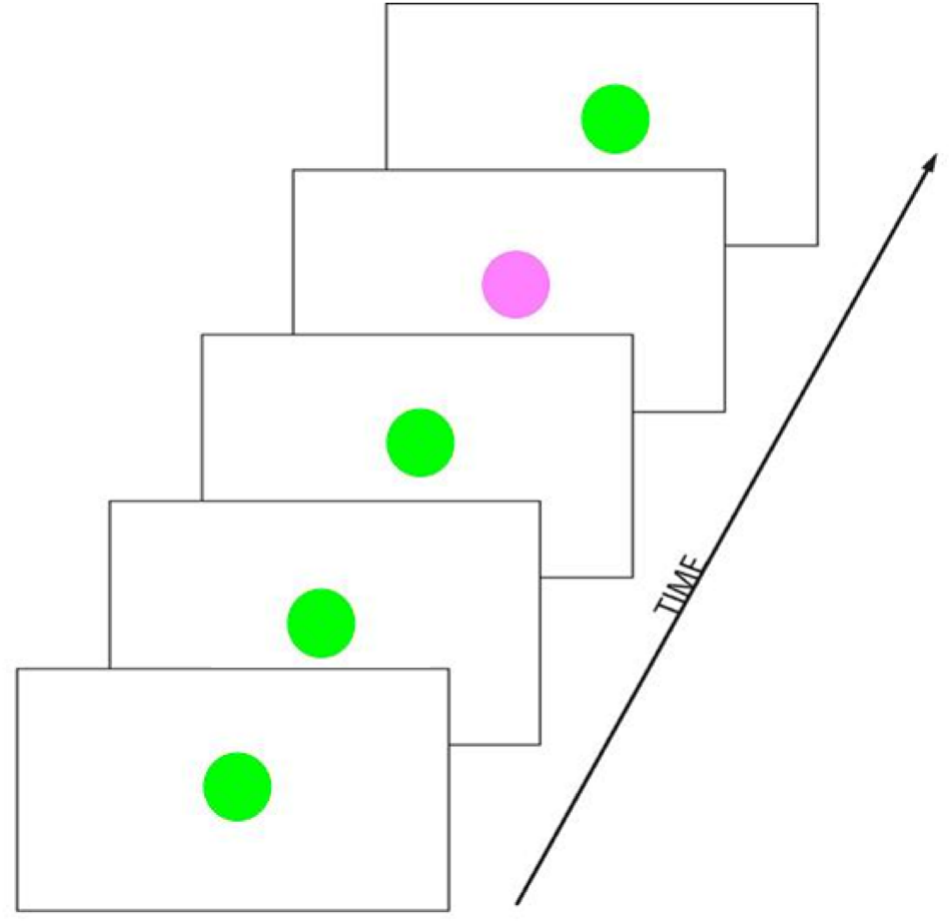
**Example of an oddball paradigm in the visual domain.** Visual stimuli are presented in a temporal sequence. The green colored circle stimulus is frequently presented and is referred to as the “frequent” or “standard” stimulus. The pink colored circle is rarely presented and is referred to as the “rare” or “deviant” or “target” stimulus. The number of standards presented between two deviants is pseudo-random.

A rather basic paradigm for testing a simple form of SL, referred to as the “Oddball” paradigm, contains a rare (or “deviant”) target stimulus presented along with more frequent (or “standard”) non-target stimuli in a serial input stream (**Figure 4**). This paradigm elicits a P300 ERP component, one of the most studied components of ERP research (for a review, see Polich, 2007). The P300 is thought to reflect a decision based on an evaluation or categorization of the stimulus. The amplitude of the P300 is highly sensitive to the stimulus probability and to the level of attention. In the oddball paradigm, the number of repetition of standards between two occurrences of a (target) deviant is randomized, such that the length of the sequence of interest is not fixed, but random. The perceiver is thought to “compute online” a conditional probability of the target occurrence. Stadler et al. (2006) were able to show how decision and preparatory mechanisms are affected by this conditional probability, by measuring the P300 and the contingent negative variation (CNV, Walter et al., 1964), respectively. In this paradigm, the target cannot be predicted by the occurrence of a given stimulus. However, as the number of consecutive standards increases, the probability of occurrence of the target increases too, which increases the likelihood of a motor response requirement, hence affecting: (1) the level of attention and/or motor decision mechanisms (as reflected by the P300), and (2) the amount of motor preparation (as reflected by the CNV). Stadler et al. interpreted their results as an indication that the level of activation of decision mechanisms indexed by the P300 were continuously increasing as the target conditional probability increased while the activation of preparatory motor mechanisms according to the CNV was much like an all-or-none phenomena.

Another well studied ERP component elicited by the oddball paradigm is the mismatch negativity (MMN), which typically is thought to reflect an automatic discrimination or echoic memory updating between the standard and the deviant stimulus (for a review, see Näätänen et al., 2012). Capitalizing on the fact that the MMN is less dependent on the level of attention than the P300, van Zuijen et al. (2006) recorded these two components simultaneously with an oddball paradigm to explore how the level of attention affects SL (more on this study in a subsequent section).

**FIGURE 5:**
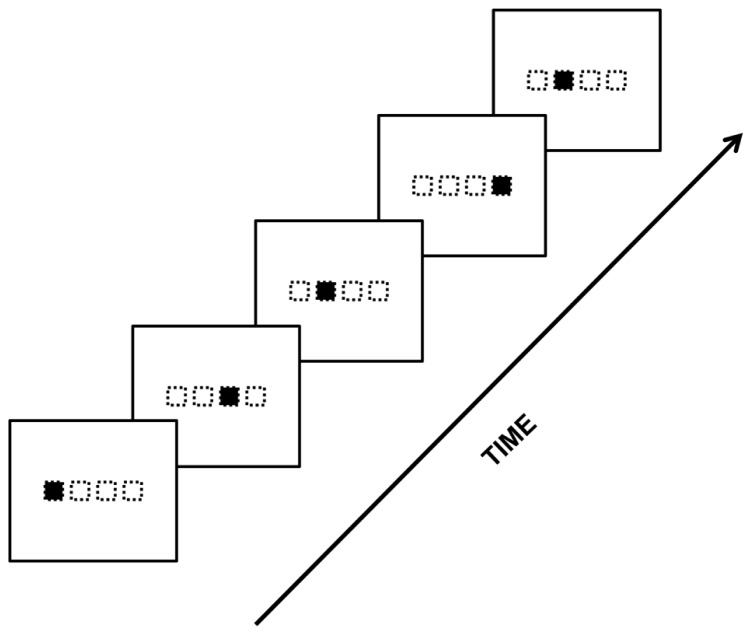
**One possible depiction of the serial reaction time task ((Nissen and Bullemer, 1987).** Visual stimuli appear at different – non-random – locations in a temporal sequence. Participants have to reproduce the displayed sequence by pressing on the touch screen at the correct locations and in the same temporal order as the displayed sequence. Note that the actual configuration of the stimulus locations can vary across studies.

Some researchers have taken the standard oddball paradigm and used it to study SL processes that occur during the serial reaction time task (SRT; Nissen and Bullemer, 1987). The typical SRT task is a visuo-motor SL task where visual stimuli appear at different locations on a screen, as described by a particular rule or pattern (**Figure 5**). Response buttons correspond spatially to each location. SL is behaviorally demonstrated by a reduced response time to repeating/familiar sequences compared to novel or random sequences. The SRT has been subsequently adopted and modified by many others for various purposes (Cleeremans and McClelland, 1991; Perruchet and Amorim, 1992; Willingham et al., 1993; Reed and Johnson, 1994; Stadler, 1995; Jiménez et al., 1996; Perruchet et al., 1997; Frensch et al., 1998; Honda et al., 1998; Reber and Squire, 1998; Shanks and Johnstone, 1999; Destrebecqz and Cleeremans, 2001). Most relevant to the present purposes, the SRT has also been used with ERP recordings, revealing ERP correlates of SL (Eimer et al., 1996; Baldwin and Kutas, 1997; Rüsseler and Rösler, 2000; Rüsseler et al., 2003a; Ferdinand et al., 2010; Meiri, 2011). Specifically, under an oddball-type version of the SRT that involves the presentation of deviant stimuli occurring in a sequence of standards, an enhancement of the N200 to deviants compared to standards has been reported (e.g., Eimer et al., 1996; Rüsseler and Rösler, 2000; Schlaghecken et al., 2000). Note that an important question is whether this modulation stems from SL *per se* or from a secondary effect of SL, for instance, an effect of attention. We will also come back to this issue in a subsequent section of this review.

**FIGURE 6:**
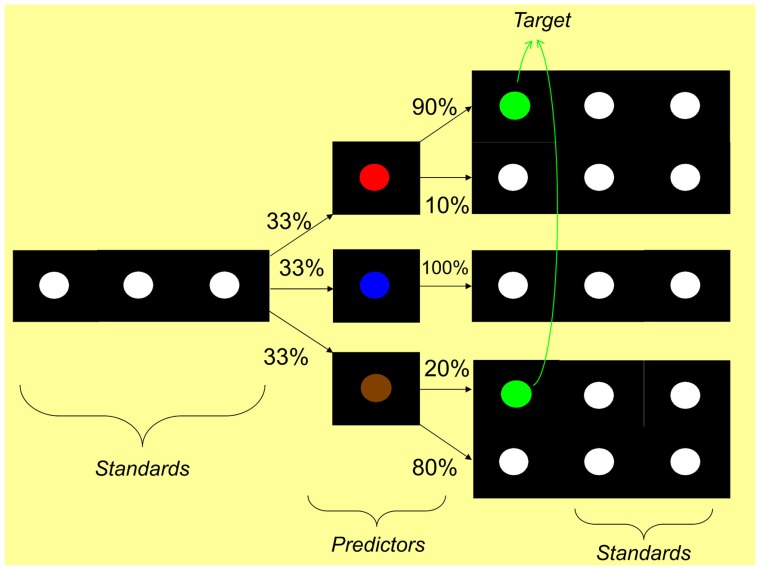
**Modified oddball paradigm of Jost et al. (2011).** The standard stimulus is a white circle on a dark background. The paradigm comprises several deviant stimuli belonging to two different categories: “predictor” and “target”. Participants are asked to press a button when the target is presented. There are three types of predictors (corresponding to the three experimental conditions): a “high probability” predictor which is followed 90% of the trials by the target, a “low probability” predictor, followed 20% of the trials by the target, and a “zero probability” predictor, which is never followed by the target. Participants are not told about these predictor-target variable statistical contingencies. SL is observed behaviorally when performance improves with higher statistical contingency. SL is observed neurophysiologically when the ERP to the predictors differ between the experimental conditions (e.g., a larger amplitude for the high probability predictor compared to the other two predictor types).

One final variation of the oddball design comes from Jost et al. (2011). This paradigm included sequences of visual stimuli (colored circles) containing a frequent stimulus and a set of “deviant” stimuli. These deviants belonged to two different categories: “predictors” and “targets” (**Figure 6**). The participant is asked to respond to target stimuli without being told that certain predictor stimuli predict the occurrence of the target with fixed contingent probabilities. That is, the occurrence of the predictor allows the participant to predict the target with varying probabilities. The assumption is that this design requires a kind of basic statistical learning of the contingent probabilities that links the predictors to the targets. Jost et al. (2011) reported a late positivity in response to the predictors between 300 and 600 ms post-predictor onset that increased as the contingent probability increased. This ERP effect was referred to as a P300-like component and interpreted as reflecting an index of SL. Similarly, Rose et al. (2001) reported an SL effect as reflected by an increased P300 to the first stimulus of a two-item sequence. According to these authors, since the task required a motor response to the second item, the ERP to the first item was also modulated by: (1) an increased lateralized readiness potential component (LRP, e.g., Hackley and Valle-Inclán, 2003), reflecting an increased motor preparation to the predictable second item (see also Eimer et al., 1996; Rüsseler et al., 2001), and (2) a decreased CNV, reflecting a reduced motor preparation to other alternative, non-predictable second items.

Unlike these oddball paradigms where the sequences embody rather simple contingent statistics, other ERP paradigms have been used to explore SL using more complex sequences, such as the “artificial grammar” paradigm.

#### Artificial grammar and natural language paradigms

**FIGURE 7:**
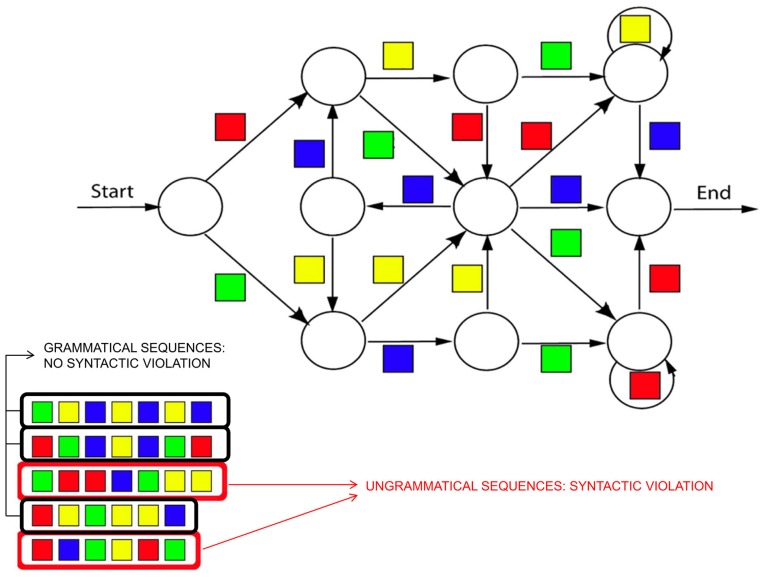
**Example of an artificial grammar in the visual domain.** The algorithm describes the rules of the artificial grammar, that is the set of possible sequences of stimuli (in this case, colored squares) that are valid according the rules of the grammar. Examples of valid sequences (i.e., grammatical sequences containing no syntactic violations) are presented on the bottom of the figure circled in dark. Examples of non-grammatical sequences (containing syntactic violations) are also presented, circled in red.

Artificial grammar learning (AGL) paradigms, which incorporate a set of rules that govern the structure of sequences (**Figure 7**), have been designed to mimic the complex structure of natural language while simultaneously removing other potentially confounding parameters such as semantic information. Converging evidence has suggested that this experimental design is a good model for testing the grammatical and structural processing of natural language (for a review see Christiansen et al., 2002). It should be noted that the AGL paradigms used in ERP research often incorporate aspects of the SRT paradigm, described above (Nissen and Bullemer, 1987). In such a combined SRT-AGL task, the structure of the sequence of stimuli follows the rules defined by an artificial grammar to determine what stimulus occurs next in the sequence.

The ERP research using AGL has shown that several ERP components known to index grammar/syntactic violation in natural language (e.g., Steinhauer et al., 2001) and in music perception (e.g., Patel et al., 1998) are also elicited by artificial grammar violations (Osterhout and Holcomb, 1992; Christiansen et al., 2012; Tabullo et al., 2013). The most commonly reported ERP indices of syntactic violation are an “early” negativity and a “late” positivity. The early negativity is usually found at left anterior cortical sites and between 200 and 400ms poststimulus-onset (but see for instance Hoen and Dominey, 2000), and hence is often referred to as the early left anterior negativity (ELAN) (e.g., De Diego Balaguer et al., 2007; Mueller et al., 2008). The late positivity, being often maximal around 600ms is usually referred to as the P600 (e.g., Steinhauer et al., 2001).

Using such AGL paradigms, it is possible for instance to test whether SL is processed by different mechanisms for different sequence structures. For instance, Bahlmann et al. (2006) reported two ERP components to grammar violation of CV syllables sequences, an early negativity within a 300–400 ms window that was evoked only by local violation [in (AB)n sequences] and a late positivity within 400–750 ms that was evoked by both local and longer range violation (in center-embedded AnBn sequences). These ERP results confirm earlier predictions of the existence of different cognitive mechanisms engaged for the processing of different types of input structures (e.g., Conway and Christiansen, 2001).

Other ERP components have also occasionally been reported as indices of SL during exposure to artificial grammars: the error-related negativity (ERN, Gehring et al., 1993), the N200, the slow negative wave (SNW), and the N400. Rüsseler et al. (2003b) used an Erikson-like flanker task wherein a central imperative letter followed a sequence or was randomly chosen and reported sequence error monitoring as reflected by the ERN. This finding suggests that the detection of (artificial) syntactic violations is cognitively processed as a specific instance of a more general set of errors, as reflected by the ERN. Lang and Kotchoubey (2000) using an AGL paradigm based on sequences of vowels within a passive task not requiring a motor response reported two frontally distributed ERP effects to rule violations: one at a latency of 250 ms – a N200 – and another around 500 ms – a SNV. Lang and Kotchoubey (2000) suggest that this SNV may in fact be an instance of the “family” of N400 components. This would be in line with other studies that also propose the N400 (Kutas and Federmeier, 2011) as an index of SL processes (Sanders et al., 2002; Cunillera et al., 2006, 2009; Carrión and Bly, 2007; De Diego Balaguer et al., 2007; Abla et al., 2008; Buiatti et al., 2009).

The AGL paradigms allow one to test SL mechanisms independently of the effects of other language processes. However, natural language paradigms remain useful even with these potential confounds, as they allow one to better understand how SL might directly contribute/interfere with language processing. The research using natural language paradigms has mainly reported an ELAN and a left anterior negativity (LAN; for an overview see Friederici, 2002) as well as a P600 (e.g., Osterhout and Holcomb, 1992) as markers of syntactic violations. For instance, Friederici et al. (2002) reported a similar P600 and ELAN to artificial and natural language grammar violations in native-speakers. This result suggests that adult who are learning a new (artificial) language use the same learning mechanisms as are used in natural language. A similar conclusion comes from Mueller et al. (2005), who reported similar ERP patterns from non-native Japanese speakers trained to learn a “Mini-Japanese” compared to native Japanese speakers. Similarly, Christiansen et al. (2012) found a P600 to syntactic violations in artificial grammars and natural language paradigms in the same set of participants. The amplitude of the P600 was correlated between the two tasks, suggesting that identical or similar underlying mechanisms were engaged in both non-linguisitc SL and natural language processing. These studies suggest that a successful methodological approach is to combine the AGL and natural language paradigms in order to more fully understand SL and natural language processing.

In summary, several ERPs components, such as the N200, the MMN, the N400, the ERN, the ELAN, the LAN, the P300, and the P600 seem to be modulated by SL in various experimental paradigms and hence may be used to better understand the cognitive mechanisms underlying SL. The variety of relevant paradigms ranges from simple sequence designs such as oddballs to more complex sequential stimuli involving natural or artificial grammars. We now consider to what extent the ERP research helps elucidate questions about the underlying mechanisms of SL and the associated representations from the perspective of the three dimensions previously discussed: the level of abstractness, the level of attention or consciousness (i.e., implicit versus explicit mechanisms), and the developmental trajectory.

### WHAT THE ERP FINDINGS TELL US ABOUT SL

#### *ERP* Findings: level of abstractness

As previously discussed, SL is thought to stem from at least two different types of mechanisms, one that acts on rather concrete information and the other that acts on more abstract information. With concrete feature-learning mechanisms, SL is explained by the encoding of distributional properties of the sequence of items, such as item co-occurrences or the transitional probability between items. The alternative (or complementary) mechanism assumes that the perceiver encodes abstract rules (or discrete combinatorial systems).

One of the crucial results from ERP studies of SL is provided by Pulvermüller and Assadollahi (2007), who attempted to dissociate ERP correlates of SL mechanisms between those that process concrete versus abstract information. To this aim, these authors manipulated separately concrete features (item co-occurrences or transitional probability) and abstract features (syntactic rules or grammaticality) of sequences using ungrammatical word strings, very rare grammatical word strings (i.e., with low co-occurrence and low transitional probabilities), and common grammatical word strings (i.e., with high co-occurrence and high transitional probabilities). Pulvermüller and Assadollahi reported a magnetic MMN that differed between grammatical and non-grammatical word strings but was unaffected by the co-occurrence (or transitional probability) manipulation. These authors concluded that natural language grammar learning would stem from the encoding of discrete combinatorial systems (i.e., abstract rules) rather than the learning of co-occurrence and/or transitional probability (i.e., concrete features). However, an alternative interpretation could be drawn from their data: both mechanisms processing concrete and abstract features might occur during syntactic processing, but the magnetic MMN could be more sensitive to abstract compared to concrete features encoding. Put another way, just because an ERP correlate was not observed for concrete feature learning does not mean that such a correlate does not exist; null effects in ERP research are notoriously difficult to interpret.

Lelekov et al. (2000) were also able to explore the issue of the level of abstractness of the information encoded during SL. Using an AGL paradigm, they presented instances of sequences of type ABCBAC and DEFEDF with different surface structure (i.e., different concrete distributional properties) but identical abstract structure. These authors reported a late positivity at 500ms, similar to the typical P600 to syntactic violation, in response to abstract structure violation, but no ERP effect to surface (concrete) structure violation. As with the Pulvermüller and Assadollahi’s (2007) study, at least two conclusions could be drawn: either only SL mechanisms of abstract structures occur or both concrete and abstract structures are processed by the mechanisms of SL but in their paradigm the ERP are mainly sensitive to those mechanisms that act on abstract information and less sensitive to those related to concrete feature encoding.

Conversely, other studies have found ERP correlates – specifically, the MMN – related to concrete feature encoding (Deouell et al., 1998; Marco-Pallarés et al., 2005; Schröger et al., 2007). For instance, Schröger et al. (2007) used standard and deviant tone pairs of different frequencies, either ascending or descending. The first tone of the pair had either a fixed frequency of 900 Hz or a random frequency within 600–1200 Hz using 10 Hz-steps. The second tone of the pair had a short or a long duration (200 or 400 ms). Schröger et al. (2007) referred to the condition with a fixed-frequency first tone as sequences with a “concrete rule” and to the condition with a random-frequency first tone as sequences with “abstract rules.” ERPs were time-locked to the second tone of the pairs. Schröger et al. (2007) reported a MMN to deviant pairs with both concrete and abstract sequences. These authors also used source localization analyses and concluded that the MMN sources elicited by abstract and concrete rule violations involved a similar neural network.

In summary, some of the few ERP studies that explored the level of abstractness of the encoded information during SL have been interpreted as evidence that SL is governed only by abstract rule-learning mechanisms. On the other end, other ERP research were taken as evidence that concrete-rule encoding can also be indiced by ERPs. Overall, it appears that the ERP research supports the assumption that both concrete and abstract feature encoding occurs in SL. The apparent inconsistency between these studies may be due simply to variation in experimental designs and a lack of sensitivity of ERP to adequately index particular mechanisms of SL.

#### *ERP* Findings: level of attention and conscious awareness

Across various paradigms, not just those specifically looking at SL, almost all ERP components have been reported to be modulated by the level of attention (Kok, 2000; Barry et al., 2003; Correa et al., 2006). Thus, one might consider the possibility that several studies that interpreted ERP components as markers of SL were in fact pointing to a (top-down) attentional effect that may or may not be specific to SL itself. For instance, Sanders et al. (2002) reported an increased N100 to learned/segmented pseudowords compared to new/unfamiliar pseudowords with exposure to a speech-like stream of unfamiliar pseudo-words. These authors concluded that the N100 is an index of SL (or segmentation). However, an alternative top-down account of this result could be that, as pseudo-words become more and more familiar due to SL (or segmentation), the pseudo-words are better recognized, and hence are more likely to capture attention. The increased N100 across exposure to a speech-like stream would thus reflect a top-down attentional effect to items of this stream. If this attentional effect is indeed occurring, an important question is whether it contributes or not to the actual process of SL (or segmentation) itself.

The literature contains several other ERP studies that attribute to ERP components the property of indexing SL while often ignoring the alternative top-down attentional explanation (Rose et al., 2001; Sanders et al., 2002; Cunillera et al., 2006, 2009; De Diego Balaguer et al., 2007; Abla et al., 2008). For instance, Abla et al. (2008) and Sanders et al. (2002) interpreted an increased N100 and N400 to segmented/learned sequences of three items [tones in Abla et al. (2008) and syllables in Sanders et al. (2002)] as reflecting the indexing of SL mechanisms. Similarly, Rose et al. (2001) reported an increased P300 with SL to the first item of a sequence of two items and several other SL studies concluded that the P200 is a marker of SL (Cunillera et al., 2006, 2009; De Diego Balaguer et al., 2007). As with the case of the Sanders et al. (2002) study, all of these ERP effects could instead be due to modulations of the level of attention, rather than SL *per se*. However, even if this top-down account is true, the ERP components still reflect an outcome of the SL process, that is, a learning-related change of attention to stimuli based on whether or not the stimuli are consistent with the previously learned patterns.

Whether SL requires conscious awareness is a hotly debated topic. The relation between implicit SL, explicit SL, and ERPs has been mostly explored through two approaches: by dissociating implicit from explicit learning according to whether participants acquired explicit knowledge of the patterns (e.g., Eimer et al., 1996; Baldwin and Kutas, 1997; Rüsseler and Rösler, 2000; Schlaghecken et al., 2000; Rüsseler et al., 2001) or by dissociating these two types of learning according to whether participants had or had not an intention to learn the rules (e.g., Rüsseler et al., 2003a,b).

In line with early behavioral studies of SL (Reber, 1967; Saffran et al., 1996, 1997), several ERP studies, using different experimental approaches, provide strong evidence that there is at least an implicit component of SL (Saarinen et al., 1992; Dell’Acqua et al., 2003; Carral et al., 2005; Kessler et al., 2005; Zachau et al., 2005; Kranczioch et al., 2006; van Zuijen et al., 2006; Trippe et al., 2007; Acqualagna et al., 2010; Yu et al., 2011; Batterink and Neville, 2013). Indeed, several ERP studies concluded that SL had occurred under conditions of minimal attention (Saarinen et al., 1992; Carral et al., 2005; Zachau et al., 2005; van Zuijen et al., 2006). For instance, van Zuijen et al. (2006) investigated the attentional issue by recording the MMN (assumed to reflect attention-independent discrimination, but see Arnott and Allan, 2002; Müller et al., 2002) and the P300 (assumed to be more dependent on the level of attention, but see Bennington and Polich, 1999). They used an oddball paradigm wherein standards are tone pairs with an ascending frequency and deviants are tone pairs with a descending frequency. Participants who after the ERP session did not report the presence of deviants, i.e., were subjectively unaware of them, showed only a MMN, while participants who were aware of the deviants showed also a P300. These findings suggest that both implicit and explicit SL can occur, each recruiting different neural mechanisms. In a study similar to van Zuijen et al. (2006), Gottselig et al. (2004) tested SL using an oddball paradigm containing eight-tone sequences [instead of tone pairs in van Zuijen et al. (2006)]. Deviant sequences differed from standard sequences only by the frequency of one tone. Similar to van Zuijen et al. (2006), Gottselig et al. (2004) were also able to record a MMN to deviants while participants’ attention was focused on silent films, thus suggesting again that implicit SL of very basic input sequences is possible.

Still using the oddball paradigm and measuring the P300, but under rapid stimulus presentation - the so-called RSVP paradigm – other studies tested the perception of a deviant within an attentional blink (Dell’Acqua et al., 2003; Kessler et al., 2005; Kranczioch et al., 2006; Trippe et al., 2007; Acqualagna et al., 2010; Yu et al., 2011). These studies concluded that there was an implicit component of SL. A similar conclusion was also reported using AGL paradigms (Baldwin and Kutas, 1997; Schröger et al., 2007) and syntactic violations within natural language (Batterink and Neville, 2013). For instance, Batterink and Neville (2013) reported early ERP deviations to such syntactic violations while the participant’s attention was focused on a distractive task. This result indicates that SL of more complex rules than those found in an oddball paradigms might also be processed implicitly.

Importantly, none of the above-mentioned ERP studies rule out the possibility that explicit mechanisms of SL also contribute to the reported ERP effects. Indeed, the ERP research on SL mechanisms has abundantly explored the explicit component(s) of SL (Tiitinen et al., 1994; Eimer et al., 1996; Baldwin and Kutas, 1997; Rüsseler and Rösler, 2000; Schlaghecken et al., 2000; Rüsseler et al., 2003a,b; Miyawaki et al., 2005; Schröger et al., 2007). For instance, Schröger et al. (2007) reported a combination of implicit and explicit SL using violations of abstract auditory rules. Standard and deviant tone pairs of different frequencies were used, in which deviant and standard pairs could have either ascending or descending frequency and the second tone of the pair had a short or a long duration (200 or 400 ms). They manipulated the effect of attention on the rules by using three conditions: (1) a passive (i.e., no task) “ignore” condition wherein participants are asked to watch a soundless video, (2) an active rules task-irrelevant “distraction” condition wherein participants were asked to perform a two alternative-forced choice discrimination decision on duration, judging whether the second tone of each pair was short or long, and (3) an active rules task-relevant “detection” condition wherein participants were asked to detect deviant pairs after having been informed of the rising/falling frequency rule. Schröger et al. (2007) not only confirmed the above-mentioned reports of an implicit component of SL showing ERP effects to deviants modulated by the participants’ performance on a non-rule related task, they also provided findings regarding the effect of the participant’s intention. Schröger et al. (2007) results suggest that intention to learn improved the ability to perform the non-rule related task. All together, these data suggest that SL can be both implicitly and explicitly learned, depending on the participants’ intention. A similar effect of the intention to learn sequences was found by Miyawaki et al. (2005). These authors presented sequences of eight digits and found that, after training, the amplitude of the N200 component (and behavioral performances in sequence free and cued recall) were higher with intention to learn compared to non-intention.

**FIGURE 8:**
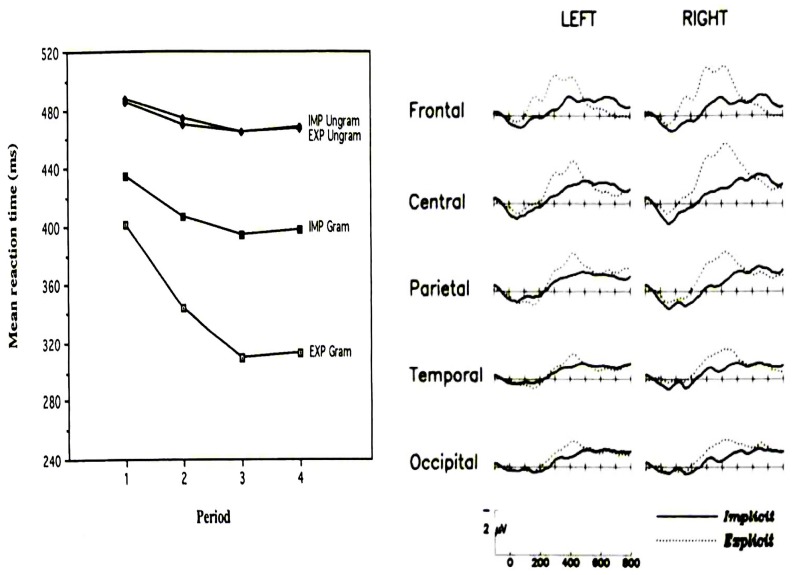
**Left panel:** Mean response time to a SRT for grammatical (“Gram”) and ungrammatical (“Ungram”) sequences across practice sessions (each session lasts for four hours) under implicit (“IMP,” participants were not previously informed of the sequence structure) and explicit conditions (“EXP,” participants were previously informed of the sequence structure). **Right panel:** Difference waves (ERP to ungrammatical targets minus ERP to grammatical targets) under implicit and explicit conditions. (Reproduced with permission from Baldwin and Kutas, 1997).

**FIGURE 9:**
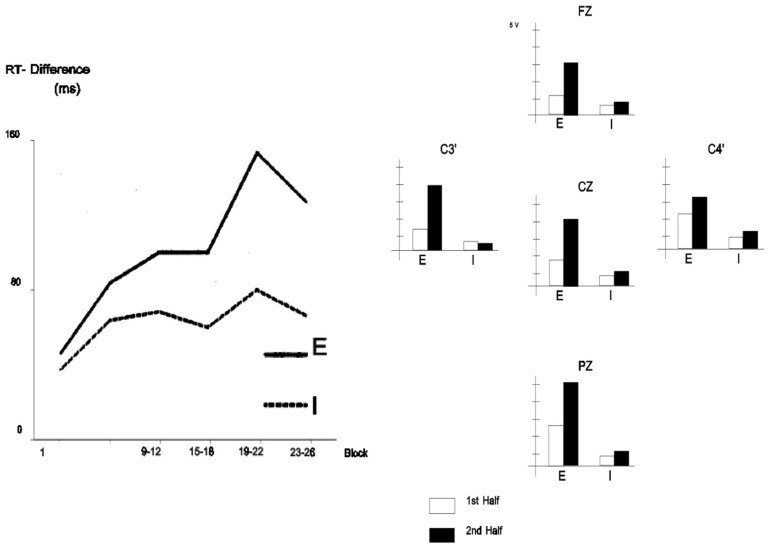
**Left panel:** Mean response time difference to a SRT (RT to ungrammatical sequences minus RT to grammatical sequences) across practice sessions/blocks (each block consists of 120 trials with the presentation of 12 sequences of 10 letters) under implicit (“I,” participants who did not report noticing the presence of a sequence when asked after the experiment) and explicit conditions (“E,” participants who reported noticing the presence of a sequence when asked after the experiment). **Right panel:** Mean ERP amplitude in the 240–340 ms poststimulus onset time range (corresponding to the N2 component) to the deviant stimulus (ungrammatical sequences) minus ERP to the standard stimulus (grammatical sequences) under implicit (“I”) and explicit conditions (“E”) from the first and second halves of the blocks. (Reproduced with permission from Eimer et al., 1996).

In addition, larger effects of learning (as measured by behavior and ERP) appear to be found in explicit compared to implicit conditions. For instance, Baldwin and Kutas (1997) provided evidence that behavioral measures of SL were roughly twice as large for explicit compared to implicit SL (**Figure 8**). In addition, these authors reported P300 effects to sequence violations that were, when explicit SL occurs, more than two times larger than those observed when only implicit SL was permitted (**Figure 8**). A similar “effect size doubling” on behavioral performance was reported by Eimer et al. (1996, see **Figure 9**) using 10-letter sequences with standard and deviant sequences. The effect size increase was even larger when measuring the amplitude of the N200. In the same vein, Rüsseler and Rösler (2000) and Schlaghecken et al. (2000), reported N200 and P300 modulations to sequence violation only in participants that learned explicitly the sequence [according to post-experimental free recall and recognition tests in the Rüsseler and Rösler’s (2000) study, and according to the “process dissociation procedure” of Jacoby, 1991 in the study of Schlaghecken et al. (2000)].

However, robust effects of explicit SL are not systematically reported. For instance, Rüsseler et al. (2003b) found similar behavioral and neurophysiological effects in implicit and explicit conditions. Rüsseler et al. (2003b) measured the ERN while participants performed an Erikson-like flanker task wherein a central imperative letter followed a sequence or was randomly chosen. The lack of difference between these conditions is likely to stem from the use of a rather unusual SL paradigm. Indeed, using a more typical SL paradigm with 16-letter-long sequences irregularly disrupted by deviant stimuli, Rüsseler et al. (2003a) were able to show a strong effect of intention on ERP effects of SL. These authors reported ERP effects on the N2b- and P3b-components only in participants who were informed of the presence of sequences and no ERP effects in a group of participants who were not previously informed of these stimulus patterns.

In summary, the ERP literature seems to support the existence of both implicit and explicit mechanisms of SL. Furthermore, the effect size of the SL measured behaviorally or neurophysiologically appears to increase with the intention to learn the rules and with the explicit knowledge of these rules. Therefore, when attempting to understand the mechanisms of SL, a very critical aspect appears to be the attentional/consciousness dimension. Importantly, since the level of attention can affect almost all ERP components, the interpretations of ERP correlates of SL must be cautious as in some instances there may be an alternative top-down explanation.

#### *ERP* Findings: developmental trajectory

In general, there is a paucity of ERP research examining SL in young children. However, neural signatures of infant and children’s early language learning mechanisms – presumably dependent in part on SL – have been documented using ERPs. Indeed, ERP studies have provided some evidence that the ability to extract statistical dependencies between adjacent elements in the speech stream appears to be present from birth, and infants can learn non-adjacent dependencies in a natural, non-native language by 4 months of age (Teinonen et al., 2009; Friederici et al., 2011). From about 9 months of age, familiar words evoke responses that are different in amplitude as well as in scalp distribution measurements from responses to unfamiliar words (Molfese, 1990; Vihman et al., 2007). By 11 months of age, phonetic learning can already be observed; by 14 months, responses to known words are observed; and by 2.5 years, semantic and syntactic learning is elicited (Kuhl and Rivera-Gaxiola, 2008). For instance, a P600 to sentence-level syntactic violations has been found in 30, 36, and 48 months old children that looked rather similar to the P600 found in young adults (Silva-Pereyra et al., 2007).

Although SL is assumed to be important for language acquisition, few studies have directly examined the relationship between SL and language outcomes. Recently, the link between SL and children’s language performance has received new support. Rosas et al. (2010) reported an ERP study of SL in children (6–11 years) using visual sequences. The authors compared two groups of children: one with and one without attention deficit hyperactive disorder. Rosas et al. found that both behavioral and ERP findings pointed to the occurrence of SL in both experimental groups. However, their most striking ERP result seems to be a considerable difference in ERP amplitude between the two groups of children on a late positivity (between 400 and 800 ms post-stimulus onset) similar to the P600, suggesting that non-linguistic SL incorporates mechanisms also used for language learning (as reflected by the P600). The fact that the two groups differed on the magnitude of the P600 also suggests that differences in attention can modulate the P600 effects to SL in children. The relation between SL and natural language is predictive from a developmental perspective, as the early mastery of the sound patterns of one’s native language provides a foundation for later language learning. Indeed, children who show enhanced ERP responses to phonemes at 7.5 months show faster advancement in language acquisition between 14 and 30 months of age (Kuhl et al., 2008).

As concerns older populations, the literature about ERP correlates of SL is scarce and mostly involves oddball paradigms that elicit, for example, the MMN and the P300 (Fabiani and Friedman, 1995; Fabiani et al., 1998; Berti et al., 2013; Cheng et al., 2013). In line with behavioral data suggesting more age-related SL deficits for structures that include long range dependencies (Curran, 1997; Howard and Howard, 1997; Feeney et al., 2002; Howard et al., 2004), MMN studies show more age-related decline with interstimulus intervals larger than 2 s (Czigler et al., 1992; Pekkonen et al., 1993, 1996; Cooper et al., 2006; Ruzzoli et al., 2012) compared to shorter intervals (Cheng et al., 2013). This decline has been interpreted in terms of faster sensory memory trace decay in the older compared to the younger adults (Pekkonen, 2000; Näätänen et al., 2007). These results suggest that the behavioral studies showing age-related decline of SL due to impaired abilities to represent and maintain context information (Braver et al., 2001) might not only stem from working memory-related deficits but also from sensory memory impairements, as reflected by the MMN attenuation. Regardless as to whether or not working memory and sensory memory share underlying mechanisms (Jääskeläinen et al., 2011), these ERP studies of aging seem to point to an age-related impairment of memory systems that might in turn affect SL ability.

Clearly, the developmental trajectory of SL still has many unexplored fundamental questions. We believe the ERP technique has not been used to explore SL across the lifespan to its fullest potential. This research gap in the developmental dimension as well as opened questions left by the previously discussed models of SL models lead us now to consider several new lines of ERP research that we believe could offer new insights into SL, some of which are amenable to developmental approaches.

### NEW DIRECTIONS FOR RESEARCH

As mentioned earlier, SL mechanisms can be explored on the dimensions of the abstractness of the manipulated representations (i.e., whether it reflects abstract rule-learning or concrete/distributional learning) and attention (i.e., the question of implicit versus explicit SL). For these two approaches, ERPs, allowing the assessment of “online” cognition, could make a nice contribution if new paradigms are applied to control for the amount of concrete information available in the input and the level of attention (or consciousness) brought to bear. In this regard, the control of concrete information could be performed using the so-called “balanced chunk strength design” (e.g., Knowlton and Squire, 1996). This procedure allows one to control for the amount of potential chunks or fragments that can emerge from the stimuli, independent of whether or not the stimuli conform to grammatical rules. As concerns the level of attention, further insights about the underlying implicit and explicit mechanisms of SL could be explored with ERPs using, for instance, the process-dissociation procedure (Jacoby, 1991). This method seems particularly promising when combined with the balanced chunk strength design and ERP, as questions such as whether chunks reflect the content of the attentional focus, or whether there exist chunks that participants are not aware of could be tested. Furthermore, it is important to attempt to tease apart the encoding of input (during a “training” phase) versus the expression of knowledge (during a “test” phase) as the level of attention may differentially impact each process (Hendricks et al., 2013). Such a line of research could be used to test the 2-step theory of Perruchet and Pacton (2006), who posited that chunks are unconsciously extracted via a bottom-up process, and then become consciously available, in a second step, for top-down processing.

We mentioned earlier that almost all ERP effects observed in SL paradigms can be interpreted either as indices of the SL process itself or as a consequence of SL, which modulates the level of attention to the learned material (be it the full stimuli, or fragments of it, i.e., chunks). Future approaches could control the level of attention using for instance subliminal stimulation (Daltrozzo et al., 2011). Finding ERP effects of SL under subliminal stimulation would rule out the alternative attentional explanation, indicating that these ERP effects are indices of SL *per se* and not indirect effects of increased attention to newly learned materials.

Another area where ERPs can be fruitfully used is to explore the nature of multisensory SL and the ways in which different subsystems of SL interact and integrate information across domains. For instance, Walk and Conway (2011) have recently proposed that SL of multisensory patterns proceeds initially via modality-specific mechanisms, and then only at a later stage of information processing, are cross-modal contingencies learned. This type of two-stage theory, in which an earlier process is posited to be followed by a later one, is perfectly amenable for exploration by ERPs, which provide a precise temporal profile of information processing. For instance, ERPs could be used to measure within-modal versus cross-modal violations in an SL paradigm, with the prediction following from Walk and Conway (2011) that cross-modal processing will occur at a later latency than within-modal processing.

In addition, future ERP research could focus more on examining the developmental time-course of SL. This issue can be assessed either on a short time scale, with for instance the analysis of the development of SL across trials within a single experiment; or on a longer time scale, with groups of participants of various ages. Both approaches have been followed for instance by Jost et al. (2011). Indeed, the short time scale approach is particularly well-suited for ERP research because it provides an online assessment of cognitive processing. At a longer time scale, the ERP technique presents also some advantages as compared to other techniques, such as behavioral measures. Whereas behavioral data, which can be rather messy to collect from children, might show a particular developmental pattern, ERP data, which can be elicited even without a behavioral response, might show an entirely different pattern of results. For example, in Jost et al. (2011), two groups of children of different ages and one group of young adults participated in an SL task while ERPs were recorded. Despite the behavioral data showing SL only in the adults group, the ERPs indicated SL also in the children.

Importantly, developmental approaches should not be restricted to comparisons between age groups. What is also needed to better explore SL at a longer time-scale are longitudinal studies (as previously suggested by Conway et al., 2011 and Arciuli and von Koss Torkildsen, 2012). The use of longitudinal studies, for instance, would help provide evidence for a causal relationship between SL and language performance. The demonstration of causality, by showing that SL at a young age predicts language outcomes later in life, would in turn have important implications for clinical intervention. So far, recent research has found a strong link at the neural level between SL and language performance using correlational research strategies (Christiansen et al., 2012; Tabullo et al., 2013). But this type of research design only allows one to conclude that there exists an association between SL and language performance, not necessarily a causal relationship.

In this manner, one potentially important way that ERPs can be used is to assess to what extent SL is amenable to cognitive or behavioral intervention. Because it has been argued and empirically demonstrated that SL is related to language performance (Conway et al., 2011; Daltrozzo et al., 2013), incorporating novel training techniques in an attempt to improve SL could have a causal impact on (i.e., transfer to) language ability (Daltrozzo et al., 2013). In this vein, using ERPs to monitor changes in SL and language abilities after receiving SL training is an important next step. Such an intervention might be even more efficient if it is combined with a biofeedback procedure. Research indicates that the combination of ERP monitoring and biofeedback shows impressive results in terms of neuronal plasticity (e.g., Miltner et al., 1986; Rosenfeld, 1990; Kotchoubey et al., 2000; Birbaumer et al., 2006).

We also suggest that additional research ought to attempt to tackle more realistic learning situations. For example, some models of SL have incorporated the interaction with the speech community and other social cues. Goldstein et al. (2010) and Tomasello (2000) have proposed models that include a bottom-up analysis of statistical regularities reinforced by a top-down attentional mechanism driven by social context cues. The influence of the social environment on SL could be accounted for by an associative memory component, or a retrospective mechanism, which facilitates processing of the stimulus (McClelland, 1979; Dale et al., 2012). According to Dale et al., SL is explained by both a predictive mechanism, as modeled by simple recurrent networks (Cleeremans and McClelland, 1991; Misyak et al., 2009), and a retrospective mechanism, which facilitates subsequent processing in a top-down manner (see also Conway et al., 2010). More research is needed to tease apart the potential role of such top-down processing in more realistic social and linguistic situations, and how this impacts SL.

Finally, it is essential that future research also recognizes the need for exploring several dimensions of SL together, because by only assessing one dimension alone, we may suffer from an overly simplistic and perhaps inaccurate view of the underlying mechanisms of SL. For instance, it might be that different aspects of SL such as the level of abstractness of the encoded representations and the level of consciousness of the learned patterns may develop along different developmental trajectories (although our proposed model predicts that these two aspects develop in parallel, **Figure 2**). As indicated earlier, ERPs are particularly well-suited to explore each of these dimensions and could also be used to explore combinatory modulations of each of these dimensions.

## CONCLUSION: AN INTEGRATIVE MODEL

SL mechanisms can be described along several partially-overlapping dimensions: the level of abstractness of the encoded sequential information, the level of attention/consciousness of these representations and the mechanisms that manipulate them, as well as the developmental trajectory. Based on these descriptors, several cognitive and computational models have been proposed. Although many disagreements and unanswered questions remain about these views, a general picture emerges. As an integrative model, we propose that SL is most likely governed by at least two types of systems whose respective contributions vary across the life-span (**Figure 2**).

In many regards, the results of the ERP research are in line with a two-systems view of SL, as opposed to just one system. However, ERP findings appear to provide inconsistent evidence with regard to the relative involvement of concrete versus abstract rule-learning components. This could be merely due to the greater sensitivity of ERPs to one or the other process and therefore the extent that ERPs are a reliable index of different mechanisms of SL. On the other hand, this might not be an intrinsic weakness of ERP but instead may point to methodological weaknesses in the assessment of consciousness, attention, and intention. To overcomes these limitations, several methodological improvements could be used in conjunction with ERP research, including the process-dissociation procedure (Jacoby, 1991) or dual-task methodology (Hendricks et al., 2013), with the aim to test the two-systems hypothesis along the dimensions of consciousness, attention, and intention. Furthermore, more nuanced ways of investigating the level of abstractness of the information encoded through SL could rely upon balanced-chunk strength designs (Knowlton and Squire, 1996).

In sum, this review has explored to what extent ERP findings can be used to better understand the neurocognitive mechanisms of SL. Rather than continuing to argue over a simple dichotomy of abstract versus concrete feature encoding or implicit versus explicit mechanisms, future research must be more aware of the potential complex relationships among multiple neurocognitive mechanisms that may differ along one or more of these dimensions based on the task at hand. Furthermore, ERPs can be used to shed light on the developmental progression of these various mechanisms. If the two-system view of SL (**Figure 2**) is correct, then this helps frame our understanding of the nature of many related aspects of cognition including motor skill development, perceptual processing, and language acquisition. One potential outcome of an improved understanding of the mechanisms of SL is the ability to design novel language rehabilitation interventions, capitalizing on the assumption that improving performance on SL could have a transfer effect and thereby improve the performance of other cognitive processes, such as language, that stem from it.

## Conflict of Interest Statement

The authors declare that the research was conducted in the absence of any commercial or financial relationships that could be construed as a potential conflict of interest.
